# Breastfeeding-oriented education for parturients separated from their hospitalized infants: a qualitative study of nurses’ perspectives in Shanghai, China

**DOI:** 10.1186/s12884-022-05227-4

**Published:** 2022-12-01

**Authors:** Haoxue Feng, Ying Liu, Junying Li, Hui Jiang

**Affiliations:** 1 Shanghai First Maternity and Infant Hospital, School of Medicine, Tongji University, Shanghai, 200092 China; 2 Obstetrics Department, Shanghai First Maternity and Infant Hospital, Shanghai, 201204 China; 3 Gynecology Department, Shanghai First Maternity and Infant Hospital, Shanghai, 201204 China; 4 Nursing Department, Shanghai First Maternity and Infant Hospital, Shanghai, 201204 China

**Keywords:** Breastfeeding, Health education, NICU, Maternal separation, Qualitative study

## Abstract

**Background:**

The benefits of breastfeeding for both mother and baby are well recognized. However, the separation of the mother-newborn dyad leads to a lower breastfeeding rate. These parturients who are separated from their hospitalized infants are sometimes unaware of the importance of breastfeeding, while nurses do know how important health education on breastfeeding is and how it can be improved. This descriptive qualitative study aimed to explore the experiences of nurses regarding health education on breastfeeding and summarize the potential ways to improve it.

**Methods:**

A descriptive phenomenological qualitative approach was utilized in this study, and in-depth, semi-structured interviews were conducted with nurses at a tertiary A-grade obstetrics-and gynecology-specialized hospital in Shanghai, China. The purposive and snowball sampling method was used and Colaizzi’s seven-step phenomenological analysis was employed. The Consolidated criteria for Reporting Qualitative research (COREQ) was followed to report findings.

**Results:**

Fifteen nurses participated in the study and shared their suggestions based on their experiences. Four overarching themes emerged from the data: (1) extending the education duration, (2) enriching the educational content, (3) expanding the education subjects, and (4) perfecting the educational process. Each theme included several subthemes.

**Conclusion:**

Health education on breastfeeding should focus on the time, content, subjects, and process as a whole. The nurses’ statements provided a reference for nursing or hospital supervisors to take measures to improve education and increase the breastfeeding rate of hospitalized neonates. Further research from the perspectives of parturients and their family members is needed, to find out what the key points are that all of them attach importance to.

**Supplementary Information:**

The online version contains supplementary material available at 10.1186/s12884-022-05227-4.

## Background

The benefits of breastfeeding (BF) for both mother and baby are well-documented in the literature. Maternal health benefits include a decreased risk of postpartum hemorrhage [1] and prolonged amenorrhea [2] in the short term, as well as lower risks of breast cancer, ovarian cancer, diabetes, hypertension, and heart disease in the long term [3]. Human milk for children is food as well as medicine, so BF promotes their growth and is also associated with a decreased risk of obesity, gastroenteritis, asthma, and sudden infant death syndrome [4, 5]. The World Health Organization (WHO) and The United Nations Children’s Fund (UNICEF) recommend that infants should be breastfed exclusively, even without any water, for 6 months and continue to be breastfed with the introduction of complementary food for up to 2 years of age or beyond [6]. The exclusive breastfeeding (EBF) rate in the first 6 months is targeted to reach at least 50% by 2025 [7]. The goal for the EBF rate in China is also over 50% by 2025 [8], while the current rate is just 29.2%, according to a survey conducted with the mothers of over 10,000 6-month-old infants [9].

With medical technology advancing rapidly, a larger number of premature or at-risk neonates have survived. However, these infants are usually admitted to the hospital for further treatment or observation, which leads to them being separated from their mothers physically and psychologically. Besides the newborn’s congenital immaturity or illness, if the mother is not in a clinical condition that allows her to stay with the baby in the same room, they have to be separated from each other. The separation of the mother-newborn dyad occurs with an incidence of 10.0-21.3% [10], which continues to increase annually. This separation hinders both the maternal-neonatal emotional bonding and attachment and the establishment of a milk supply [11, 12]. As hospitals in countries like China usually don’t allow mothers to stay with their baby when they are admitted to a neonatal intensive care unit (NICU), BF is always encountering challenges. The extraction of breast milk is hence an expedient [13].

For hospitalized infants, the benefits of human milk are even more pronounced. The risk of infection, short- and long-term health morbidities, and mortality is higher in those who fail to receive human milk with immunological properties [14]. The BF rate of hospitalized infants, which is only 3.7-20.0%, reportedly, is comparatively lower than that of healthy babies in China [15]. Elevating the BF rate of the special neonates is highly valued while challenging. Faced with their babies being in medical need all of a sudden, many families have made little preparation for caring, and must learn it before discharge. Neonatal intensive care unit staff and former NICU parents all identified with the importance of health education, which is the key to successful care during hospitalization and after discharge [16, 17]. Despite the consensus that maternal separation requires the parturients to express their milk to send it to their babies, many of them don’t realize the importance of doing this, or don’t know how to do it, even if they are willing to. One of the critical missions of health care professionals in the NICU is to equip the parents with enough information in a relatively short time.

While there has been attention to mothers’ experiences BF a hospitalized preterm infant [18], little attention has been paid to the status quo of BF among hospitalized infants from a professional perspective. Nurses in the obstetric department or a neonatology clinic, the main characters who do the health education, are equipped with sufficient knowledge and experience as they are engaged in front-line work almost every day. From the nurses’ perspective, they have a clear view of what the mothers or families are suggested to know, what strengths and weaknesses the health education on BF has, and what can be done to achieve further refinement. Therefore, this qualitative study interviewed nurses working in the obstetric department or a neonatology clinic, aiming to learn about their experiences with health education on BF, and ask for advice to improve it. We require knowledge about the challenges when nurses do health education to improve the BF rate of hospitalized neonate, and how to improve the effect of health education on BF more efficiently from a relatively professional perspective. The findings of this study were aimed at providing references for nursing or hospital supervisors to take measures to increase the BF rate of hospitalized infants in the future.

## Methods

### Design

A descriptive phenomenological qualitative approach was utilized in this study. Descriptive phenomenology originated from Husserl’s (1960) work and is commonly used in nursing and midwifery research [19]. Descriptive phenomenology is a specific school of phenomenological psychology, which aims to describe the essential structure of a specific experience [20]. In Husserl’s descriptive approach, researchers are required to focus on participants’ experience of a phenomenon and identify the essences of the phenomenon while suspending their own beliefs, attitudes, previous experience and assumptions [21]. The Consolidated Criteria for Reporting Qualitative Research (COREQ) was used to report findings (see Additional file 1) [22].

### Setting

This study was conducted at Shanghai First Maternity and Infant Hospital, a tertiary A-grade obstetrics-and gynecology-specialized hospital in China, which is affiliated with Tongji University and joined the Baby-Friendly Hospital Initiative in 1992. Every year, about 30,000 babies are born in the hospital. The hospital holds pregnancy schools; once registered, expectant mothers can take a course free of charge. If her fetus is diagnosed with some defect or complicated condition in prenatal examinations, the woman will be offered a “one-stop” diagnosis-therapy service by the Fetal Medicine Department, which follows the philosophy of seeing the “fetus as a patient.” After delivery, if the infant is considered to be at-risk, they will be admitted to the NICU inside the Neonatology Clinic. There are also several out-patient clinics for discharged mothers and infants, like Jaundice Clinic and High-risk Neonatal Follow-up Clinic. If the parturients face difficulties breastfeeding, the International Board-Certified Lactation Consultants (IBCLCs) at the Breastfeeding Department can help them; if their breast duct is plugged, they can turn to the Milk Duct Clearing Department; and if they have little idea how to take care of their babies, or whether the babies’ responses are normal or not, the Home Care Department is a good choice. In China, there is a tradition “zuòyuèzi”, or “sitting/doing the month.” Parturients have to stay home and rest for a “confinement period” of about 1 month (or 42 days) after giving birth, which is thought to facilitate recovery. During this period, a maternity matron (yuesao) may be hired to take care of the new mother, and help with household and childcare tasks [23].

### Researchers and positionality

All of the team members were female registered nurses with experience in descriptive qualitative research and a background in breastfeeding research. All interviews were conducted by the first author HF, studying for a master’s degree. She had no child of her own or experience with breastfeeding, which wouldn’t lead to a bias. JL was studying for a master’s degree as well, without a child. YL, a doctoral student, had one two-year daughter and successful experience in breastfeeding. HJ, an experienced qualitative researcher, who had an adult child, provided specialist methodological support frequently.

### Ethical consideration

Ethical approval was obtained by the Ethics Committee of Shanghai First Maternity and Infant Hospital before conducting the research (No. KS20238). The study was performed in accordance with the Declaration of Helsinki and followed the institutional guidelines. All participants were informed of the purpose and confidentiality of the study. Every participant explicitly consented to the study and to be recorded while being interviewed. Confidentiality was ensured by not disclosing any participant names or personal information. All of the audio recordings and transcripts were coded and saved on a password-protected computer. Nurses who agreed to participate were allowed to withdraw at any time without any consequences.

### Sampling and participants

The purposive and snowball sampling method was applied [24]. To be specific, the interview began with the head nurses of the Obstetric Department and Neonatology Clinic, and afterward, they recommended several other nurses in line with the inclusion criteria. Inclusion criteria consisted of (1) having worked in the Obstetric Department or Neonatology Clinic of Shanghai First Maternity and Infant Hospital for more than 5 years; (2) having an education level of a bachelor’s degree or higher; (3) being articulate and easy to communicate with; (4) being willing to be interviewed; (5) having never been involved in medical disputes; and (6) volunteering to participate in this study. After interviewing thirteenth nurses, no more new information emerged; another two nurses were interviewed to ensure data saturation [25]. None of the nurses refused to participate or dropped out.

### Data collection

Each in-depth, semi-structured interview was held face-to-face in the head nurse’s office or retiring room without anyone else present; some nurses at the Neonatology Clinic were interviewed in the breastfeeding room, which was quiet and unused at the time in consideration of COVID-19. The participant’s sociodemographic data were collected before the interviews were conducted. After that, they were asked several questions, like “What challenges do you encounter when doing health education on breastfeeding to these special mothers?” “Do you feel that these mothers benefit from your health education?” and “What kind of training on health education should be increased or enhanced?”. These questions were revised with two nursing experts and pilot tested with three nurses. The duration of the interviews ranged from 30 to 60 min based on the participant’s willingness to share. Two participants were interviewed twice for clarification and addition to their data because of their undefined responses. The whole course of each interview was recorded by cellphone. Field notes were written during and immediately after each interview.

### Data analysis

All of the interviews were conducted in Mandarin Chinese, and the recordings were transcribed verbatim within 24 − 48 h after each interview for coding purposes. Transcripts were returned to six participants because the others were so busy that they refused to read them carefully. Those who read through the transcripts didn’t report any mistake or make changes. The transcripts were managed and analyzed with NVivo 12 software, a Computer-Assisted Qualitative Data Analysis Software (CAQDAS) [26]. Two authors coded the data. If there emerged different opinions, the third author would be asked to reach a consensus on final decisions. Colaizzi’s seven-step phenomenological analysis was employed to identify the themes and subthemes. This method provides a rigorous analysis, with each step staying close to the data [27]: (1) familiarization by reading all of the transcriptions collected carefully and repetitively; (2) identifying pertinent important or meaningful statements and creating memos; (3) formulating meanings for recurring ideas and coding them; (4) clustering preliminary themes by looking for common coded ideas; (5) describing the themes exhaustively and selecting appropriate statements as quotations; (6) producing and refining themes and subthemes; and (7) seeking verification from the interviewed participants. (Six participants were randomly chosen for verification in this study.) The final step helped the authors modify earlier steps in the analysis in light of the feedback. To ensure the trustworthiness of the study, double-coding, member checking, bracketing and peer debriefing were conducted [28, 29].

## Findings

In total, fifteen nurses participated in this study. All of the nurses were female and ranged in age from 29- to 50-years-old. Twelve nurses reported having a bachelor’s degree, while the other three had acquired a master’s degree. More than half of the participants had worked as a nurse for 11 years or longer (*n* = 9). Most of the nurses worked in the Obstetric Department or the Neonatology Clinic doing a job concerning breastfeeding for 6–10 years (66.7%). General characteristics of the participants are presented in Table 1.

**Table 1:** Characteristics of the nurses interviewed (*N* = 15)

	N	%
Sex		
Male	0	0
Female	15	100
Age		
26 − 30 years	4	26.7
31 − 35 years	5	33.3
36 − 40 years	2	13.3
41 − 45 years	3	20.0
46 − 50 years	1	6.7
Education level		
Bachelor degree	12	80.0
Master degree	3	20.0
Work experience as a nurse		
6 − 10 years	7	46.7
11 − 15 years	3	20.0
16 − 20 years	3	20.0
21 − 25 years	1	6.7
26 − 30 years	1	6.7
Work experience related to breastfeeding		
6 − 10 years	10	66.7
11 − 15 years	2	13.3
16 − 20 years	2	13.3
21 − 25 years	1	6.7

Through Colaizzi’s 7-step approach, the qualitative study extracted four overarching themes from the nurses’ experience of BF-oriented education, namely “extending the education duration,” “enriching the educational content,” “expanding the education subjects,” and “perfecting the educational process.” Each theme included several subthemes: the subthemes for “extending the education duration” were (1) prenatal preparation and (2) postpartum follow-up. The ones for “enriching the educational content” were (1) lactation mechanism, (2) hand-expression skills, and (3) myths and rumors. The ones for “expanding the education subjects” were (1) husband who is the key, (2) other family members who are important as well, and (3) a yuesao who seems professional. The ones for “perfecting the educational process” were (1) content standardization, (2) constant repetition, and (3) educational training or practice for nurses (Table 2).

**Table 2:** Themes and subthemes

Themes	Subthemes
Extending the Education Duration	Prenatal preparation
	Postpartum follow-up
Enriching the Educational Content	Lactation mechanism
	Hand-expression skills
	Myths and rumors
Expanding the Education Subjects	The husband who is the key
	Other family members who are important as well
	A yuesao who seems professional
Perfecting the Educational Process	Content standardization
	Constant repetition
	Educational training or practice for nurses

### Theme 1: Extending the education duration

The first theme, “extending the education duration,” considered the long duration of the perinatal period, so health education should be started before delivery, but not end immediately upon discharge. “Prenatal preparation” and “postpartum follow-up” are suggested to become nursing routines.

#### Prenatal preparation

Nurses agreed that there was a lot of information online, and with the service of pregnancy school, it might be more effective to give prenatal care more publicity.

*“There are so many parturients with their babies to attend, let alone those who are separated from their infants. What we need to do is more than ‘breastfeeding.’ It would be more efficient to emphasize just key points if they have been equipped with related knowledge.” (P11)*

Although prenatal education is useful and helpful, most of the time, it is challenging.

*“How to mention it? If you talk about the separation after birth, won’t she be shocked about it? You can’t swear at them, can you? Most mothers are separated from their babies all of a sudden. Only if their fetus was determined to be abnormal, or at risk of preterm birth, with the possibility of being hospitalized or referred, our Fetal Medicine Dept. would hold a consultation. For these pregnant women, they are prepared to learn and listen to your suggestions, in which way their compliance and anxiety will get better afterward. I think these special mothers-to-be are our new target, and we intend to move forward on the work as well. (P12)*

#### Postpartum follow-up

Parturients are usually discharged before their babies. When they stay at home or somewhere near the hospital, most mothers choose to send their breast milk to the Neonatal Department.

“If the amount of breast milk is small, we will make a phone call to ask for further details. But, you know, sometimes the feedback is not that timely, because we are so busy that we can only arrange for some nurse to make the calls once a week, unless the baby is too severe [of a case] to accept any formula except human milk. Then we will teach them how to express milk, and what the frequency is, again.” (P10)

“For those who don’t send breast milk, we will ask them why, like ‘is it because you didn’t express the milk, or just because you didn’t send them here.’ Even if their milk isn’t consumable due to drug use, or it is not convenient for them to send milk, we will tell them to keep expressing their milk, whether discarding it or storing it in refrigerators.” (P4)

After the infants are discharged, they return back to their mothers. Bottle-feeding at the hospital makes it relatively hard to latch onto the breast, for which reason, follow-up guidance is of great significance. However, it is currently random and doesn’t cover all subjects.

“Actually, like babies treated for jaundice, we don’t follow up with them after discharge. Neither do we ask them how the breastfeeding is going. It is compulsory to follow up only with high-risk babies. For others, the obligatory target is 30% every month. So, the mothers are randomly chosen and educated.” (P8)

“Not even one baby should be omitted. Otherwise, whether it can get breastfed is basically up to luck.” (P12)

### Theme 2: Enriching the educational content

Nurses are supposed to offer an all-round education content on breastfeeding, involving the “lactation mechanism,” “hand-expression skills,” and “myths and rumors.”

#### Lactation mechanism

The extraction of breast milk timely and regularly after delivery is a determining factor in the success of breastfeeding. Nurses are always talking the parturients into expressing milk, while they seldom tell them the reason, which induces incomprehension.

“Our education content focuses on how to collect, store and send their milk, rather than on why to express the milk.” (P3)

“For her, it’s hard to understand, like ‘Why should I send my milk to you? The doctor said my baby had to fast; so why?’ or ‘You told me my baby just ate 1 ml per meal, 8 ml a day in total, so who will you give it to if I expressed so much milk?’ They don’t know the lactation mechanism, and why she should do this in the first two days.” (P1)

Their incomprehension may also lead to a vicious cycle.

“Only if the mother kept milking, the brain could make a reflex response to the stimulus. But they think their breasts haven’t produced milk yet, so they just give up trying. Why bother, right?” (P8)

“They don’t know what to do when their breasts feel ‘full.’ That’s why they do nothing at all. They fail to express milk at a regular time, or to massage their breasts. Sometimes, they think there is no need to express milk at night because they have already done it regularly in the daytime.” (P7)

“They don’t know what the consequence is, but they won’t produce enough milk when they really need it.” (P1)

#### Hand-expression skills

Without infants’ sucking, expressing milk is a necessary replacement. There are two ways for the parturients to express their milk, namely hand-expression or machine-pumping. Considering that the former is indispensable, especially in the first postpartum days, nurses will introduce the related skills to all of them, though the learning effect is far from satisfactory.

“Actually, I think sometimes, what we need to teach is no longer the importance of expressing the milk, but how to succeed in doing it.” (P12)

“I feel that many of them don’t know how to express their milk by hand. They just lack the skill, you know. And, at last, the milk will be blocked in there.” (P5)

“When in need, she may find she is incapable of hand-expression, and nobody is gonna help her, and then she will get really anxious.” (P1)

“Some mothers feel that hand-expression is painful, but they couldn’t figure out whether they were doing it right or not.” (P3)

#### Myths and rumors

In today’s information age, the parturients are always overloaded with various kinds of ideas, from the Internet or friends and neighbors. Some traditional Chinese cultures like zuòyuèzi also subtly influence them. This ancient tradition, like a natural order, means that mothers should be confined at home, and do or not do a series of things during the first postpartum month (or 42 days). Nurses ought to do pertinent education while keeping this in mind.

“The seniors living together find it essential to supplement nutrients after delivery, so they incline toward a greasy diet, which makes the milk thick and hence easy to get blocked.” (P5)

“Once we know [that] the mother’s breast duct is plugged, we will suggest her to visit our Milk Duct Clearing Dept. But many mothers don’t think they will go out during the special period. So, how can we help them?” (P1)

“There is the tradition of ‘zuòyuèzi,’ so mothers refuse to leave the home. After the baby returns back to their mom, they will try feeding at the breast. If the mother fails to breastfeed, like in a month, and doesn’t turn to our Breastfeeding Dept. for guidance, her breast milk is doomed to dry up.” (P9)

“Some mothers-in-law are always intervening. For example, they would tell the mother that breast milk is bad. Especially when the mother’s periods return, the elderly will probably ask her to stop breastfeeding, since they think the breast milk is no longer healthy. Horrible rumors!” (P1)

### Theme 3: Expanding the education subjects

Pregnancy and delivery are not about mothers only but is also about everyone around them. Therefore, the number of education subjects should be expanded, and more attention should be paid to the “husband, who is the key,” “other family members, who are important as well,” and a “yuesao, who seems professional.”

#### The husband who is the key

Husbands are the main caregiver during the perinatal period. They are the key to mothers sticking to breastfeeding because their support is the most meaningful and their ideas and behavior matter. Health education has already been offered to them, but is still not enough.

“When admitted to the hospital, health education is primarily offered to fathers because mothers are so tired in bed after the delivery or in pain due to a cesarean section. If the husband didn’t attach importance to breastfeeding, the mother probably won’t think it important after being conveyed information to. And if the husband isn’t that reliable, the information the mother received might be more problematic.” (P10)

“In the process of conveying information, since it’s impossible for the husband to remember 100% what we say, there is always something mistaken or omitted. What they care about most is their baby’s condition, rather than how to express or send human milk, so many mothers don’t even know about it.” (P4)

To express the milk to be sent is real, hard work. The husband should be aware of it and be encouraged to lend a hand.

*“The mother has to get up in the middle of the night, pump the milk, wash, and disinfect everything. Without husband’s help, they are less likely to persist.” (P5)*

#### Other family members who are important as well

As paternity leave in China is relatively short, many parturients are looked after by other family members, who will have various aspects of support, breastfeeding included.

*“The elderly in the family may question the quality and nutrition of the breast milk given its light color, praising the formula given in the hospital, and persuading the mother not to bother sending her own milk… Sometimes, it’s the mother’s own mother who is heartbroken seeing her daughter keep expressing milk around the clock. Pumping deprives the mother of enough rest, and makes her haggard and her nipples swell. As her mom, she will worry so much that she may advise [her that] a little milk is enough to stop. (P1)*

Some parturients’ mothers-in-law may put themselves in a stronger position, imposing their values upon their daughters-in-law. Unfortunately, some values they believe to be true and experienced are actually in need of correction.

*“In the first days, the baby just comes back, they will probably refuse the mother’s nipple. Some mothers-in-law would consider the mother short of breast milk, wouldn’t wait for the mother to try, or would sneakily bottle-feed the baby with formula. Some may suggest that formula is not bad since her son grew up healthily with formula as well. (P5)*

#### Yuesao who seems professional

The convention of “zuòyuèzi” prevails. Accordingly, a special population called “yuesao” has emerged, known as maternity matrons trained to take care of parturients and their infants, from breastfeeding to daily life. With regards breastfeeding, there is no professional qualification certificate for non-medical personnel confirmed by the country yet. Therefore, whether the information yuesao provide is reliable or correct is still in question.

*“The yuesao they find are not necessarily professional; you know, not necessarily qualified. Well, I feel that those yuèzi centers in the market are somewhat complicated.” (P9)*

While the mothers are still in the hospital, they can be served by the yuesao cooperating with the hospital, who are trained for the position. Relatively reliable, they should be on the list of education subjects as well.

*“We should educate our own yuesao to make sure their breastfeeding knowledge is up-to-date, and their hand-expression skills are right. They have a lot of contact, so I hope they can pass on the right information to the mothers.” (P12)*

### Theme 4: Perfecting the educational process

The success of health education is not only about its rich content but also about how the education is implemented. Therefore, the educational process needs perfecting through “content standardization,” “constant repetition,” and “educational training or practice for nurses.”

#### Content standardization

Although personalized education is highly praised in modern nursing, the educational content on average problems is yet to be standardized. For example, if parturients receive different answers from different nurses to the same question, they will be very confused.

“All [of] our nurses will educate the mothers, so [in] the education method or content, I think, there must exist bias. We have a PowerPoint on health education, but does everyone conform to it? We also have a booklet on breastfeeding, but can everyone explain it beautifully? I doubt it.” (P6)

“Maybe we can arrange for one nurse to give the education, well, to keep the consistency. If 60 nurses go do it, there will probably emerge 40 versions of educational content. When the mother or family members ask us about something out of the education booklet, we may answer based on our own experience or knowledge. We have made a PowerPoint on health education, but there is not one about breastfeeding in special.” (P4)

#### Constant repetition

Slim chances are that the parturients and their significant others can remember everything the nurses have said to them only once. Education is just like an examination; it requires constant review to obtain a satisfactory result.

“Well, our health education is complete only on admission. After that, we will make a phone call once a week while the baby is still in hospital. But, in fact, many babies are discharged within one week, so their parents haven’t enough access to professional information. They may not pay attention to breastfeeding, and no wonder they can’t persevere…. For these mothers separated from their babies, if someone keeps droning on about breastfeeding every day when they stay in the Obstetrics Dept., like a novice chanting, I believe they will realize its importance.” (P10)

“Maybe it’s useless, but, you know, better doing [it now] than never!” (P8)

Repetition can be realized verbally, and we can also increase the methods of health education.

“I think we can play some videos about breastfeeding on the TVs in our ward. In one way, it will be easier for them to understand what we tell them after watching the videos. In another way, it can deepen their good impression of breastfeeding.” (P8)

“We should do more about the education. One or two ways are far from enough. Like the booklets we deliver, you know, they don’t remember to read them. Sometimes, they are just seen as reference work. They browse them just when they need them. It is a must to stress the importance of breastfeeding in different aspects.” (P3)

#### Educational training or practice for nurses

Nurses are professionals in breastfeeding and equipped with sufficient knowledge. However, how to impart everything to the parturients in an effective way is a big issue.

“The training in education techniques can be enhanced. We do have a lot of professional knowledge, but how can we make them realize the significance of breastfeeding in such a short time? I think our skills in teaching or talking require improving. It’s really important…. How to transform the professional information into something easy to understand, and meanwhile, vivid enough that parents are willing to pay attention to it? If we can’t, it’s in vain, as it means we fail to apply the theory to practice.” (P10)

“Sometimes, because we are busy, we have to finish the education quickly. When we speak too fast, they may not be able to fully understand. It’s worth our reflection indeed.” (P2)

## Discussion

This qualitative study focused on four aspects critical to improving health education on BF from 15 nurses’ perspectives, focusing on the time, content, subjects, and process. More often than not, several of the four themes were related and interwoven together, as has also been reported in previous studies.

The period from conception to the child reaching the age of age two is a key phase, which not only affects the physical and cerebral development during the first years of life but also has an impact on the health in adulthood [30]. Thus, it is far from enough if health education on BF begins only after delivery. Pregnancy is often seen as a critical teachable time, during which women gradually realize the effects of their behaviors on their own and their baby’s health [31]. Although not every expectant mother may be ready to learn about BF when they are separated from their baby, in consideration of their psychological state and mood, as mentioned in the result, those with a high risk of postpartum separation can be identified and educated ahead of time. A smartphone-based prematurity education app named Preemie Prep for Parents (P3) was developed for pregnant women with a high risk of preterm birth and has managed to increase parent knowledge [32, 33]. At the Children’s Hospital of Philadelphia, if a woman’s pregnancy is prenatally diagnosed with a fetal anomaly, she will be offered a group prenatal care based on the CenteringPregnancy model [34, 35], where she can get support and education on BF. After discharge, postpartum education is suggested to be continued as well. A meta-analysis demonstrated that postpartum home visits could improve BF knowledge and skills effectively so as to increase the EBF rate further [36].

Other than personal choices, many mothers don’t practice BF due to a lack of knowledge or incapability to do so [37]. Inability to express human milk proves to be one of the major factors affecting BF in early postnatal period [38]. Only when they realize the reason and importance of expressing their breast milk and learn how to do it right will they become proactive. However, despite their subjective willingness and efforts, myths and rumors are somewhat influential. There is no denying that some practices of the tradition of zuòyuèzi are beneficial to postpartum recovery, while others are controversial and tiring to follow [39]. For example, activity restriction makes sure of sufficient rest, but at the same time, it reduces the frequency of expressing breast milk. Excessive intake of meat and soup can lead to excess weight and breast problems, like plugged ducts [40]. Going outside is also believed to be a taboo for fear of getting a cold or headache, which may be beneficial in a way, as sunlight promotes vitamin D production [41]. So, nurses can inform them that if their breast condition needs professional aid, they shouldn’t be confined in the room alone. Many of these common practices of zuòyuèzi come from a yuesao and older generations, like one’s mother, mother-in-law, and other relatives (e.g., one’s grandmother), who share similar perspectives and experiences [42, 43]. Because they play an important role in highly praising the tradition, nurses are suggested to add them to their education subject list. The husband, as the partner of the woman, the father of the baby, the son of the mother-in-law, and the bridge of the newly extended family, ought to be on the list as well. They also tend to acquire information directly from medical staff, rather than from their wives indirectly in case of potential misinterpretations [44]. The gap between traditional practices and contemporary evidence about the effects of zuòyuèzi upon BF and parturients’ wellbeing is required bridging; it is necessary for nurses to emphasize the positive aspect of traditional practices and debunk myths and rumors that are unnecessary, wrong, or even harmful [42].

Whether when, what, or to whom the health education on BF will take place, the way or method of doing it should be emphasized. Standardization is the premise of education. The NICU Family Support (NFS) Core Curriculum, a standardized education program, was implemented for parents of infants in NICUs; the project found that, among 3,648 attendees at 41 sites across the country, 77% of them reported learning “a lot” and 85% reported boosted confidence, with higher satisfaction and a positive knowledge change [45]. Similarly, another study also found consistent information provided through standardized discharge tools could alleviate parents’ mental stress and build up their confidence [46]. Health education on BF can also be carried out through various methods for the purpose of emphasis and effectiveness. Education materials, like paper-based posters or pamphlets, which are inconvenient to carry around, are not actually that practical or efficient [47]. Provision of educational information through mobile health (mHealth) technology is on the rise, and it has been shown that this promising technology may enhance parents’ capability to understand and apply complex information on BF, even in the event of preterm birth [33, 48]. However, if nurses’ preservice education on BF is limited to only a few hours of training, it must be insufficient to provide effective support for the mothers and families [49]. Thus, in-service continuing education (CE) is a worthy opportunity to further improve nurses’ knowledge, attitudes, skills, and practices towards BF; a 1-year follow-up study has demonstrated its long-term effectiveness and found that most nurses preferred training through an e-learning approach, while they preferred a traditional face-to-face course only in one case (positioning, latch-on, and effective suckling) [50].

Since receiving human milk is crucial for hospitalized infants, relevant measures should be adopted to increase the BF rate as soon as possible, with reference to the findings of this qualitative study. In regions where BF-oriented education isn’t paid enough attention to or needs improving, like China, nursing or hospital supervisors are suggested to assume the leading role. For example, they can encourage more healthcare providers to get trained and qualify as IBCLCs, who demonstrate specialized knowledge and clinical expertise in BF [51]. With more professional medical staff in BF emerging, the public awareness of BF can be heightened, which will make education easier and more effective. However, evidence has shown that inappropriate marketing of breast-milk substitutes is one major global barrier to BF [52]. While the promotion of breast-milk substitutes using unethical marketing practices continues throughout the world, more countries should fight back [53]. There is an urgent need to ensure full compliance with international and local regulations in future advertisements and to develop a better supervision mechanism [54]. With the creation of a positive, social environment for BF, more pregnant women, parturients, husbands and other caregivers will be willing to learn about it and get educated.

### Limitations

The main limitation of this study was the sampling method. There were a couple of IBCLCs at this specialized hospital, but only two of them were recruited. Although the data was saturated, some surprising and meaningful statements or themes might emerge if more IBCLCs were interviewed; after all, they were more professional and experienced in breastfeeding among this special population. The perspective of a “nurse” was a feature of the study, but it was also a limitation. What the nurses thought important in health education on breastfeeding was not necessarily important or essential for the parturients and their family members. Future studies should focus on the thoughts of parturients, as they are the ones who actually breastfeed. Their family members may also be interviewed to help identify better interventions for this group. A mixed-methods design in qualitative research, which can help to achieve impartiality, is expected in the future. The use of both quantitative techniques and qualitative techniques can bring balance and hence impartiality to the qualitative research.

## Conclusion

This qualitative study has identified four major themes regarding health education on breastfeeding, which also highlighted four aspects of how the effects of breastfeeding-oriented education for parturients separated from their hospitalized infants can be improved. The education should start from pregnancy and continue even after discharge, during which period nurses should provide the woman and her husband, family members, and the yuesao, with information, including on the lactation mechanism, hand-expression skills, and correct any myths and rumors. To perfect the entire education process, nurses should make sure of content standardization and repetition, and keep training and practicing. Further research from the perspectives of parturients and their family members is needed, to find out what the key points are that all of them attach importance to.

## Supplementary Information

**Additional file 1.** COREQ (COnsolidated criteria for REporting Qualitative research) Checklist.

## Data Availability

The data supporting the conclusions of this article are not publicly available because further research remains ongoing but are available from the corresponding author on reasonable request.

## Abbreviations

BF: Breastfeeding
EBF: Exclusive Breastfeeding
NICU: Neonatal Intensive Care Unit
IBCLC: International Board-Certified Lactation Consultant
